# Pharmacokinetics of Anthraquinones from Medicinal Plants

**DOI:** 10.3389/fphar.2021.638993

**Published:** 2021-04-15

**Authors:** Dongpeng Wang, Xian-He Wang, Xiongjie Yu, Fengjun Cao, Xiaojun Cai, Ping Chen, Minglun Li, Yibin Feng, Hongliang Li, Xuanbin Wang

**Affiliations:** ^1^ Laboratory of Chinese Herbal Pharmacology, Oncology Center, Renmin Hospital, Hubei University of Medicine, Shiyan, China; ^2^ Biomedical Research Institute, Hubei Key Laboratory of Wudang Local Chinese Medicine Research and School of Pharmacy, Hubei University of Medicine, Shiyan, China; ^3^ Department of Radiation Oncology, University Hospital, LMU Munich, Munich, Germany; ^4^ School of Chinese Medicine, The University of Hong Kong, Hong Kong, China

**Keywords:** anthraquinones, pharmacokinetics, Chinese medicines, natural products, medicinal plant

## Abstract

Anthraquinones are bioactive natural products, some of which are active components in medicinal medicines, especially Chinese medicines. These compounds exert actions including purgation, anti-inflammation, immunoregulation, antihyperlipidemia, and anticancer effects. This study aimed to review the pharmacokinetics (PKs) of anthraquinones, which are importantly associated with their pharmacological and toxicological effects. Anthraquinones are absorbed mainly in intestines. The absorption rates of free anthraquinones are faster than those of their conjugated glycosides because of the higher liposolubility. A fluctuation in blood concentration and two absorption peaks of anthraquinones may result from the hepato-intestinal circulation, reabsorption, and transformation. Anthraquinones are widely distributed throughout the body, mainly in blood-flow rich organs and tissues, such as blood, intestines, stomach, liver, lung, kidney, and fat. The metabolic pathways of anthraquinones are hydrolysis, glycuronidation, sulfation, methylation/demethylation, hydroxylation/dehydroxylation, oxidation/reduction (hydrogenation), acetylation and esterification by intestinal flora and liver metabolic enzymes, among which hydrolysis, glycuronidation and sulfation are dominant. Of note, anthraquinones can be transformed into each other. The main excretion routes for anthraquinones are the kidney, recta, and gallbladder. Conclusion: Some anthraquinones and their glycosides, such as aloe-emodin, chrysophanol, emodin, physcion, rhein and sennosides, have attracted the most PK research interest due to their more biological activities and/or detectability. Anthraquinones are mainly absorbed in the intestines and are mostly distributed in blood flow-rich tissues and organs. Transformation into another anthraquinone may increase the blood concentration of the latter, leading to an increased pharmacological and/or toxicological effect. Drug-drug interactions influencing PK may provide insights into drug compatibility theory to enhance or reduce pharmacological/toxicological effects in Chinese medicine formulae and deserve deep investigation.

## Introduction

**FIGURE 1:**
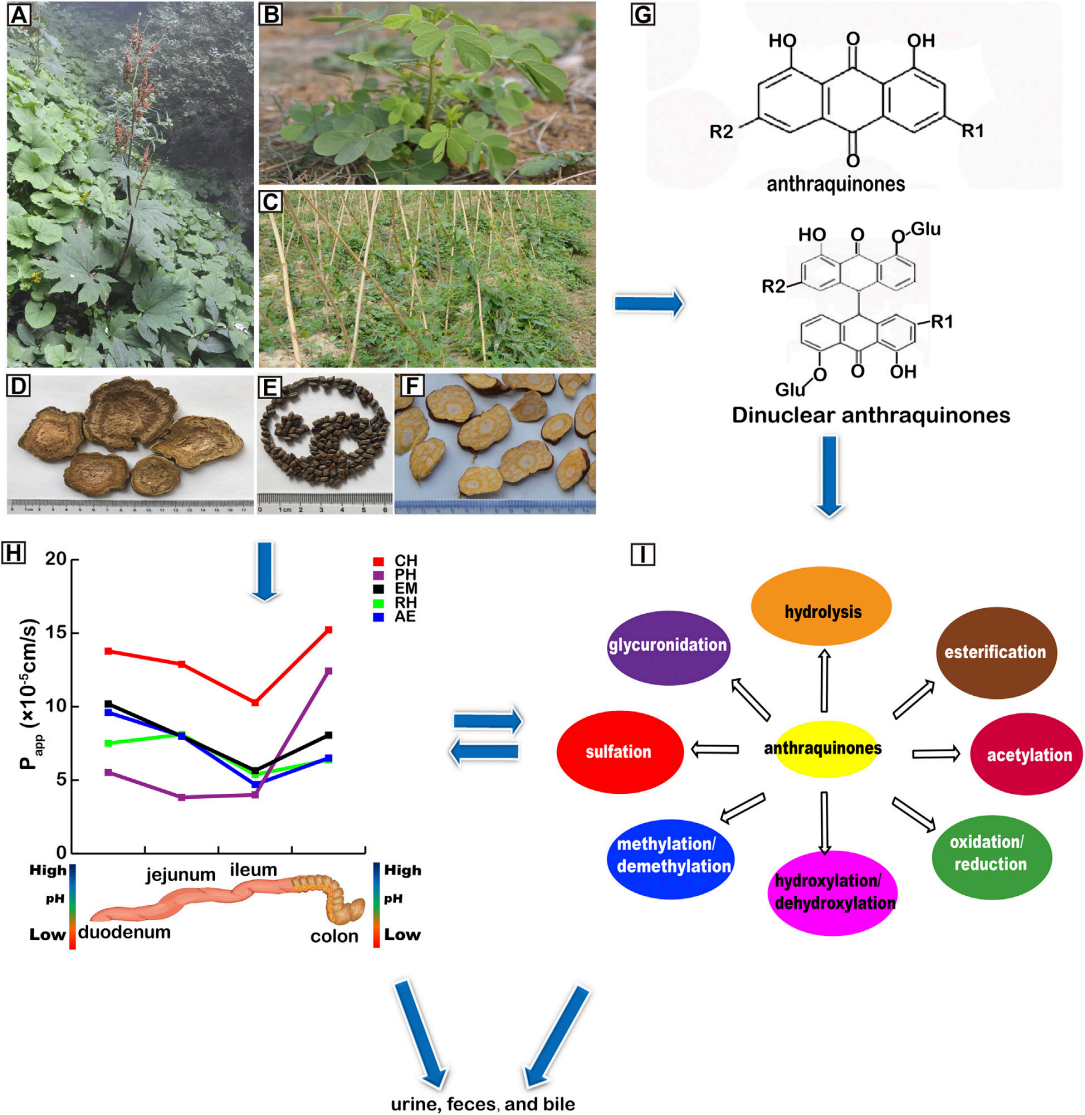
Pharmacokinetics (PK) of anthraquinones. **(A)** *Rheum officinale* Baill. **(B)** *Cassia tora* L. **(C)** *Polygonum multiflorum* Thunb. **(D)** Rhei Radix et Rhizoma. **(E)** Cassiae Semen. **(F)** Polygoni Multiflori Radix. **(G)** structures of anthraquinones and dinuclear anthraquinone glycosides. R1 and R2 represent different groups including glucoses. **(H)** absorption of anthraquinones. *P*_app_: apparent absorption coefficient. CH: chrysophanol; PH: physcion; EM: emodin; RH: rhein; AE: aleo-emodin. **(I)** metabolism of anthraquinones.

Anthraquinones naturally exist in plant families, such as, *Polygonaceae*, *Leguminosae*, *Rubiaceae* (Chen et al., 2020), *Rhamnaceae*, *Scrophulariaceae*, *Liliaceae*, *Verbenaceae* and *Valerianaceae* (Zhao et al., 2011), e.g., *Rheum palmatum* L., *Rheum tanguticum* Maxim, ex Balf., *Rheum officinale* Baill., *Cassia obtusifolia* L., *Cassia tora* L., *Verbena officinalis* L*.*, *Polygonum multiflorum* Thunb., *Aloe barbadmsis* Miller., *Aloe ferox* Miller, *Rubia cordifolia* L., *Cassia angustifolia* Vahl, *Cassia acutifolia* Delile*,* and *Morinda angustifolia* Roxb (Chen et al., 2020). Anthraquinones are also found in the secondary metabolites of lower-order plants, such as, lichens (Solárová et al., 2020). Some plants have been used for Chinese medicines (Yang et al., 2016; Li H. et al., 2018; Yang et al., 2018) (Figures 1A–F).

Pharmacological studies have shown that anthraquinones exert purgative (Gong et al., 2015), anti-inflammatory (Li D. et al., 2013), immunoregulation (Abu et al., 2018), antihyperlipidemia (Wang et al., 2014), and anticancer effects (Lin et al., 2015; Cui et al., 2016; Yang N. et al., 2019). Thus, pharmacokinetics (PKs) has attracted increasing attention and in-depth research for scholars, especially in the field of Chinese medicines.

Anthraquinones are structurally divided into two classes, mononuclear and dinuclear. Their names and CAS numbers are listed in Supplementary Table S1. The parent rings of anthraquinones are illustrated in Figure 1G.

Thanks to advanced technologies and methodologies, the pharmacological and/or toxicological effects of anthraquinones have been gradually uncovered. However, there has been no overall review of their PKs untill now, which are closely associated with their bioactions. Thus, this study summarized the PKs of anthraquinones, aiming to provide basic knowledge for further research on the pharmacological and toxicological effects and mechanisms of anthraquinones.

## Absorption

### Absorption Sites and Rate

The absorption of anthraquinones depends on their physical and chemical properties, especially quinone structure and liposolubility under the normal conditions. The dominant absorption sites for anthraquinones are the intestines rather than the stomach (Wang J. et al., 2011; Liu X. et al., 2011; Wang P. et al., 2011), although emodin is absorbed more quickly in the stomach than in the intestines (Kong et al., 2011). This may result from anthraquinones having more retention time in the intestines than in the stomach (Kong et al., 2011).

Regarding the intestines, the accumulated absorption rates of total anthraquinones in the small intestines and colons of male SD rats at 2 h are 66.99 and 23.54%, respectively (Liu X. et al., 2011). Anthraquinones can easily enter small intestinal villi epithelial cells through passive diffusion (Li et al., 2012). This can be calculated via their absorption rate constant (*K*_a_) and apparent absorption coefficient (*P*_app_) (Table 1) (Figure 1H). A larger *K*_a_ means a shorter T_max_, i.e., faster drug absorption. A larger *P*_app_ means a larger area under the curve (AUC). Actually, the *P*_app_ of anthraquinones is the greatest in the duodenum and then decreased in the jejunum and are minimum in the ileum (Qiu et al., 2011; Wang J. et al., 2011; Wang P. et al., 2011). However, the *K*_a_ and *P*_app_ of anthraquinones increased in the colon than that in the ileum (Figure 1H). This may be associated with the weak acidity of anthraquinones and the pH conditions in the intestines. Since the upper small intestines are a weakly acidic environment (Wang J. et al., 2011; Wang P. et al., 2011), given that most anthraquinones are weakly acidic, this may lead to lower ionization and higher liposolubility of anthraquinones. In contrast, with a higher pH value, the ileum is an alkaline environment (pH = 7–8), where the ionization degree of anthraquinones is increased leading to little anthraquinone absorption. However, compaired with the ileum, the *K*_a_ and *P*_app_ of anthraquinones increase in colons because the acidity increases slightly and the retention time is prolonged (Table 1).

**TABLE 1:** *K*_a_ and *P*_app_ values of some anthraquinone compounds absorbed in different intestines and colons of rats.

	Duodenum		Jejunum		Ileum		Colons		References
	*K*_am_ (×10^−4^/s)	*P*_app_ (×10^−5 ^cm/s)	*K*_a_ (×10^−4^/s)	*P*_app_ (×10^−5 ^cm/s)	*K*_a_ (×10^−4^/s)	*P*_app_ (×10^−5 ^cm/s)	*K*_a_ (×10^−4^/s)	*P*_app_ (×10^−5 ^cm/s)	
AE	5.43–16.07	7.65–10.68	4.88–13.03	6.29–9.83	2.23–8.63	3.45–5.90	3.88–12.17	5.12–7.9	(Wang et al., 2011a; Wang et al., 2011b; Qiu et al., 2011)
CH	19.02	13.77	15.15	12.88	10.80	10.27	18.17	15.22	
EM	15.55	10.18	11.45	7.98	8.38	5.65	12.45	8.05	
PH	10.08	5.53	6.38	3.83	6.22	4.00	16.12	12.42	
RH	6.96–10.68	6.15–8.91	5.70–11.13	7.95–8.22	4.79–6.27	4.17–6.59	5.18–6.55	3.85–8.92	

*K*_a_: absorption rate constant; *P*_app_: apparent absorption coefficient; AE: aloe-emodin; CH: chrysophanol; EM: emodin; PH: physcion; RH: rhein. The number of male and female rats in the studies was equal.

Generally, anthraquinones are absorbed with over a wide range *in vivo*. This may be due to differences in drug dosages, detection instruments, and protocols. As described in Table 2, the greater the body weight of the subjects is, the greater the *C*_max_ and AUC are. Among anthraquinones, rhein has the lowest T_max_, and the highest *C*_max_ and AUC in dogs (Zhu et al., 2006) (Table 2).

**TABLE 2:** The pharmacokinetic parameters of anthraquinones in rats and dogs.

Pharmacokinetic parameters	*C*_max_ (μg/ml)		*T*_max_ (h)		AUC0-∞ mg/(L·h)		References
	Rats	Dogs	Rats	Dogs	Rats	Dogs	
Aloe-emodin	0.004–124.40	0.03–0.45	0.20–11.33	0.75–1.55	0.008–4.67	0.42–1.61	(Yang et al., 2012; Zhang et al., 2013b; Feng et al., 2013; Feng et al., 2014; Jiang et al., 2015; Yang et al., 2019a; Cheng et al., 2020)
Alizarin	0.25	–	0.98	–	1.64	–	(Gao et al., 2018)
Aurantio-obtusin	0.17–1,135.80	–	0.08–0.53	–	0.99–5.90	–	(Zhang et al., 2014; Yang et al., 2015; Guo et al., 2017; Yang et al., 2019a)
Chrysophanol	0.001–3,142.80	0.04–0.30	0.25–9.28	1.00–2.00	0.01–37.05	0.54–0.83	(Yang et al., 2012; Feng et al., 2014; Zhu et al., 2017; Ullah et al., 2018; Yang et al., 2019a; Cheng et al., 2020)
Chrysophanol-8-O-β-D-glycoside	0.03	–	2.00	–	0.158	–	(Ullah et al., 2018)
Chryso-obtusin	0.05–894.1	–	0.08–3.64	–	0.27–3.58	–	(Zhang et al., 2014; Yang et al., 2019a)
Citreorosein	0.149	–	0.19	–	0.134	–	(Cheng et al., 2020)
Emodin	0.001–348.4	0.27–0.48	0.10–8.94	0.75–1.42	0.004–39.6	1.38–4.05	(Song et al., 2009b; Yang et al., 2012; Feng et al., 2014; Zhu et al., 2014; Jiang et al., 2015; Zhu et al., 2017; Yang et al., 2019a;
Emodin-8-O-β-D-glycoside	0.02–0.10	–	0.28–0.29	–	0.014–0.084	–	(Zhang et al., 2018b; Cheng et al., 2020)
Munjistin	0.03–0.74	–	1.61–1.93	–	0.14–3.99		(Gao et al., 2016; Gao et al., 2018)
Obtusifolin	0.10–1,535.5	–	0.13–3.94	–	0.24–18.17	–	(Yang et al., 2015; Guo et al., 2017; Yang et al., 2019a)
Obtusin	0.12–802.0	–	0.33–1.13	–	0.36–7.07	–	(Zhang et al., 2014; Yang et al., 2019a)
Physcion	0.03–0.49	0.03	0.17–10.4	2.00	0.07–3.29	0.48	(Feng et al., 2013; Feng et al., 2014; Huang et al., 2014; Yang et al., 2015; Feng et al., 2017; Zhu et al., 2017)
Physcion-8-O-β-D-glycoside	0.019–0.021	–	0.26–0.75	–	0.084	–	(Ullah et al., 2018; Cheng et al., 2020)
Purpurin	0.07–0.21	–	1.61–1.64	–	0.24–1.55	–	(Gao et al., 2016; Gao et al., 2018)
Questinol	0.001	–	4.38	–	0.017	–	(Cheng et al., 2020)
Questin	0.028–0.056	–	0.17–0.23	–	0.22–0.26	–	(Guo et al., 2017)
Rhein	0.001–134.0	1.44–3.39	0.08–12.00	0.71–1.50	0.002–63.14	4.24–35.15	(Yang et al., 2012; Feng et al., 2014; Jiang et al., 2015; Li et al., 2017b; Yang et al., 2019a; Cheng et al., 2020)
Xanthopurpurin	0.06	–	1.3	–	0.34		(Han et al., 2020)
1-desmethylobtusin	0.11	–	0.5	–	0.54	–	(Zhang et al., 2014)

*C*_max_: peak concentration; *T*_max_: peak time; AUC: area under the curve.

### Affecting Factors

#### Physiological Conditions

*Experimental Animal Species* The absolute bioavailability (F) of rhein in beagle dogs is higher than that in rats (49.7 vs. 23.8%, *p <* 0.01) (Zhang et al., 2010) (Table 1).

*Sex* The AUC values of emodin (Liu W. et al., 2011) and aloe-emodin (Yang et al., 2010) in male rats are higher than those in female rats. In contrast, the AUC of rhein in healthy women is higher than that in men. Furthermore, the *T*_max_ of rhein is shorter in women than that in men (Zhu et al., 2006), indicating a faster absorption of rhein in female (Yang et al., 2010). These findings may result from the difference in the body fat ratio between females and males (Zhu et al., 2006).

*Hepato-Intestinal Circulation and Reabsorbing* The blood levels of aloe-emodin, chrysophanol, emodin, chrysoobtusin, physcion-8-O-β-D-glucoside, chrysophanol-8-O-β-D-glucoside, obtusin, aurantio-obtusin, obtusifolin, physcion and rhein fluctuate dramatically due to the hepato-intestinal circulation (and glycoside hydrolysis in the intestines) (Ullah et al., 2018; Yang B. et al., 2019). Another factor is that anthraquinones are rapidly distributed to other organs and re-absorbed into the blood. Thus, aurantio-obtusin, obtusin, chrysoobtusin, emodin, chrysophanol, rhein and aloe-emodin form second absorption peaks. For example, the second absorption peaks for emodin from different studies range from approximately 3–10 h (Wu W. J. et al., 2017; Wang L. et al., 2017; Ullah et al., 2018; Yang B. et al., 2019; Zhang et al., 2019).

*Food* Compared with the fasted rats, the *C*_max_ and AUC of rhein and emodin increase in the fed group (Gong et al., 2011). However, the mechanism is currently unknown.

#### Disorders

The AUC values of rhein, aloe-emodin, chrysophanol, emodin and physcion increase significantly in the rats with microcirculation disturbance compaired with the normal group (Zhu et al., 2017). The *C*_max_ and *T*_max_ of chrysophanol are increased in acute pancreatitis in dogs compaired with the normal group (Yang et al., 2012). Conversely, rhein had a lower AUC in liver-injured male rats. The potential mechanism may result from changes in the expression and activation of metabolic enzymes in the injured liver (Zhang et al., 2015). For constipated rats, oral administration of rhubarb extract (anthraquinone-rich containing plant) resulted in a the *C*_max_ and AUC of emodin that were approximately ten times those of normal rats, while the AUC values for aloe-emodin and rhein were decreased. The mechanisms may be attributed to the direct action of aloe-emodin and rhein on intestinal cell membranes and the indirect action of emodin on bowel movement through adjustment by the nervous system (Gong et al., 2015). This may synergistically enhance the purgative effect on constipation.

#### Drug–Drug Interactions

Drug–drug interactions always alter the single herb pharmacological effects. Since natural products especially Chinese medicines are always used as formulae that consist of two or more herbs, they play a critical role in investigating the influencing factors of drug-drug interactions in PKs. Generally, the combination of anthraquinones with other drugs has three types, pure compounds of anthraquinones, anthraquinone-containing single herbs (including their extracts and fractions), and anthraquinone-containing herbs in formulae.

Anthraquinone-containing single herbs combined with other drugs or single-herbs (herb pairs) are a basic building block for Chinese medicine use. For example, a rhubarb-gardenia herb pair consisting of Rhei Radix et Rhizoma (Dahuang containing anthraquinones) (Figures 1A,D) and Gardeniae Flos (Zhizihua, containing genipin) is used for treating cholestasis diseases. A study showed that Gardeniae Flos increased the *C*_max_ and AUC of aloe-emodin, chrysophanol, emodin and rhein in rats, indicating a synergistic effect of the rhubarb-gardenia herb pair on hepatoprotection (Dong et al., 2015).

Compared with pure compounds and single herbs, interactions between herbs in a formula are the most common to assess drug compatibility for traditional Chinese medicines.

Da-Cheng-Qi decoction (DCQD), a classic formula including Rhei Radix et Rhizoma (“monarch” herb), Magnoliae Officinalis Cortex (Houpo), Aurantii Fructus Immaturus (Zhishi), and Natrii Sulfas (Mangxiao, Na_2_SO_4_·10H_2_O), has been used for treating acute pancreatitis and intestinal obstruction. Combining DCQD with ranitidine (an H2 receptor inhibitor) is a Chinese-Western integrative strategy for such diseases. Thus, it is necessary to investigate the drug-drug interactions between ranitidine and DCQD. Ren et al. reported that ranitidine reduces the *C*_max_ and AUC of rhein in DCQD. Therefore, the bioavailability of DCQD may be decreased, indicating the dosage of DCQD should be increase when combined with ranitidine. This may result from ranitidine changing gastrointestinal motility and inhibiting the absorption of rhein. (Ren et al., 2009).

San-Huang tablets, consisting of Rhei Radix et Rhizoma, extracts of Scutellariae Radix (Huangqin) and berberine hydrochloride, are used for multiple diseases, such as constipation, inflammation, pathogenic microbes, and spasm. Rhei Radix et Rhizoma is the main component for constipation because of its active compound, emodin. Studies have shown that Scutellariae Radix and/or berberine hydrochloride increased the AUC and *C*_max_ of emodin, indicating a potentiation role of Scutellariae Radix and/or berberine hydrochloride in the efficacy of emodin (Zhou et al., 2010). Moreover, Xin et al. reported that San-Huang-Xie-Xin decoction (SHXXD), including Rhei Radix et Rhizoma, Scutellariae Radix and Coptidis Rhizoma (containing berberine), increases the *C*_max_ and AUC of rhein compared with a single herb of Rhei Radix et Rhizoma (Xin et al., 2009). The mechanisms may be due to the inhibited glucuronidation activity of UDP-glucuronyltransferases (UGTs) by other ingredients in SHXXD, leading to the increased bioavailability of rhein (Hou et al., 2014).

Dahuang-mudan decoction (DMD) consists of Rhei Radix et Rhizoma, Moutan Cortex (Mudanpi), Persicae Semen (Taoren), Benincasae Semen (Dongguaren), and Natrii Sulfas. DMD has been used for treating intestinal carbuncles for approximately 1,700 years since the Han Dynasty. Pharmacological effects on appendicitis, inflammatory bowel disease, pelvic inflammatory disease and acute pancreatitis have been found with the identification of active compounds, emodin, aloe-emodin, rhein, paeoniflorin and amygdalin. Nong et al. reported that Natrii Sulfas decreases the *C*_max_ and AUC of emodin and rhein while increasing the absorption of aloe-emodin, indicating novel insight into the role of Natrii Sulfas in DMD in addition to a stool softener treatment of archenteric inflammatory disease (Zhang Y. X. et al., 2013; Nong et al., 2019).

Tao-He-Cheng-Qi-Tang (THCQT), including Persicae Semen (Taoren), Rhei Radix et Rhizoma, Natrii Sulfas, Cinnamomi Ramulus (Guizhi), and Glycyrrhizae Radix et Rhizoma (Gancao), has been used to treat platelet aggregation, hyperlipidemia, diabetes, inflammation, and related conditions. Xie et al. reported that compared with the oral administration of Rhei Radix et Rhizoma alone, the *C*_max_ and AUC of rhein in THCQT increased in rabbits. However, the mechanisms for the alternation of rhein absorption are unknown (Xie et al., 2005).

An eight-herb formula, Niu-Huang-Jie-Du tablets (NHJDT), including Bovis Calculus (Niuhuang), Rhei Radix et Rhizoma, Realgar (As_2_S_2_, Xionghuang), Gypsum Fibrosum (CaSO_4_·2H_2_O, Shigao), Platycodonis Radix (Jiegeng), and Borneolum Syntheticum (D-borneoland, Bingpian), exerts heat clearance and detoxification in Chinese medicine. Compaired with oral adminstraton of Rhei Radix et Rhizoma alone in rats, the AUC and *C*_max_ of rhein increased in NHJDT, while the *T*_max_ of the chrysophanol isomer decreased. The mechanism requires further study (Liu Y. et al., 2018).

## Distribution

### Tissues and Organs

Since the bioavailability of anthraquinones is low, to date, only a few distribution studies of the anthraquinones aloe-emodin, chrysophanol, emodin, rhein, and physcion have been reported, as listed in Table 3. These anthraquinones are widely distributed and are more abundant in blood-rich tissues and organs, such as the intestines, stomach, plasma, lung, liver, heart, and kidney. More intestine and stomach distribution may facilitate anthraquinone treatment of digestive gut disorders. They are also detected in fat, possibly due to their good liposolubility. However, few anthraquinones have been discovered in the brain since they have difficulty passing through the blood-brain barrier (Ding et al., 2003; Shia et al., 2011b; Tan et al., 2013; Chen et al., 2014; Du et al., 2014), although chrysophanol easily enters the brain when it is prepared in liposomes (Zhu et al., 2012).

**TABLE 3:** Distribution of anthraquinones in various tissues and organs.

Components	Species/biomatrix	Administration routines	Administration dosage	Distribution	References
Aloe-emodin	KM mouse	i.g.	300 mg/kg (rhubarb extract)	Intestines, stomach, kidney, lung, muscle, liver, heart, fat, brain, plasma, spleen	(Wang et al., 2020)
Aloe-emodin	KM mouse	i.g.	52.2 mg/kg, 26.1 mg/kg, 13.05 mg/kg	Intestines, heart, lung, liver, kidney, brain, stomach, spleen, muscle, fat, plasma	(Li and Feng, 2018)
Chrysophanol	KM mouse	i.g.	300 mg/kg (rhubarb extract)	Stomach, intestines, liver, spleen, kidney, fat, lung, plasma, muscle, heart, brain	(Wang et al., 2020)
Chrysophanol	New Zealand rabbits	i.v.	15 mg/kg	Heart, lung, liver, kidney, brain	(Tan et al., 2013)
Chrysophanol	SD rats	i.g.	15 mg/kg	Heart, kidney, spleen, liver, lung, brain	(Chen et al., 2014)
Chrysophanol	KM mouse	i.v.	10 mg/kg	Blood, heart, kidney, spleen, liver, lung, brain	(Zhu et al., 2012)
Emodin	KM mouse	i.v.	(5.45 μg,13.7 nmol) 0.1 ml	Blood, lung, kidney, stomach, thyroid, liver, bone, small intestines, skin, heart, spleen, mucle, brain	(Du et al., 2014)
Emodin	KM mouse	i.g.	300 mg/kg (rhubarb extract)	Stomach, intestines, liver, kidney, lung, spleen, plasma, fat, heart, muscle, brain	(Wang et al., 2020)
Rhein	KM mouse	i.g.	300 mg/kg (rhubarb extract)	Liver, stomach, intestines, plasma, spleen, kidney, lung, heart, fat, muscle, brain	(Wang et al., 2020)
Rhein	SD rats	i.g.	2.0 g/kg of rheum palmatum L. decoction	Kidney, liver, lung	(Shia et al., 2011b)
Physcion	KM mouse	i.g.	300 mg/kg (rhubarb extract)	Intestines, stomach, liver, lung, spleen, heart, plasma, muscle, fat, brain, kidney	(Wang et al., 2020)

### Affecting Factors

#### Physiological Condition

*Sex* After oral administration of 4.5 mg/kg of ^14^C-aloe-emodin to rats, the concentration of aloe-emodin in rat ovaries is higher than that in testes (Lang, 1993). The amounts of emodin and rhein in the liver of female rats are greater than those in male rats (Chen et al., 2017). The different distribution between males and females suggests that sex should be taken into consideration before clinical drug use.

#### Disorders

The distribution of anthraquinones in tissues and organs is associated with therapeutic target sites, effects and storage. More tissue distribution may involve stronger efficacy on tissues and organs. Regarding gastrointestinal diseases, aloe-emodin, rhein, rhein-8-O-β-D-glycoside and sennoside A are distributed at higher levels in the liver and colon in the constipation model mice than in the normal group when they are treated with a Chinese formula, Dahuang-Gancao decoction. The greater distribution in the colon may benefit the treatment of constipation (Chen et al., 2019). For acute pancreatitis, rhein in Da-Cheng-Qi decoction is distributed more in the pancreas than in normal rats, indicating a promising effect of Da-Cheng-Qi decoction on acute pancreatitis (Zhao et al., 2015). To investigate the potential change in the distribution of rhubarb anthraquinones, the total extract of Rhei Radix et Rhizoma was orally administered to normal and CCl_4_-induced liver injury rats. Data have shown that the distribution of aloe-emodin, emodin and rhein in the rat spleen, liver and kidney is decreased under liver injury (Fang et al., 2011), which deserves further study. The distribution of anthraquinones is listed in Table 3.

## Metabolism

Biotransformation is an important process for anthraquinones to be changed into inactive or more active metabolites and cleared from the body. The transformation occurs mainly in the liver. However, since most Chinese medicines are orally administered, biotransformation of anthraquinones has already begun in the early phase of absorption in the gut based on the actions of enzymes in the intestinal flora, including *Bifidobacterium* sp. (Wang et al., 2010), *Peptostreptococcus*, *Clostridium* spp., and *Eubacteria* (Rong et al., 2016). (Table 4 and Figure 1I).

**TABLE 4:** Metabolic pathways and metabolites of anthraquinones.

Compound	Animal species	Dose	Administration routines	Metabolic pathway	Metabolites	References
Aloe-emodin	SD rats	10 mL/kg rhubarb decoction	i.g.	Glucuronidation, hydroxylation, hydrogenation, oxidation	Aloe-emodin-8-O-glucoside-1-O-glucuronide or aloe-emodin-1-O-glucoside-8-O-glucuronide, 2-hydroxyaloe-emodin-ω-O-glucuronide	(Song et al., 2010)
Aloe-emodin	SD rats	NA	Liver microsomes	Monohydroxylation, hydrogenation, methylation, oxidation in side chain	Aloe-emodin, rhein, 1,8-dihydroxy-3-hydroxymethyl-10-oxanthranol, 1,2,8-trihydroxy-3-hydroxymethylanthraquinon, 1,4,8-trihydroxy-3-hydroxymethylanthraquinon, 1,8,9,10-tetrahydroxy-3-(methoxyl)methyl-9,10-dihydroanthracene, 1,8-dihydroxy-3-(methoxyl)methylanthraquinone, 1,8-dihydroxy-3-hydroxymethyl-4-methylanthraquinone, 1,8-dihydroxy-3-hydroxymethyl-2-methylanthraquinone	(Song et al., 2009a)
Aloe-emodin	SD rats	0.035 mg/mL	Liver microsomes	Hydroxylation, reduction, oxidation	Dihydroxy-aloe-emodin, hydroxy-aloe-emodin, hydroxy-rhein, hydroxyl-1, 8-dihydroxy-3-hydroxymethyl-9-oxanthranol/hydroxyl-1, 8-dihydroxy-3-hydroxymethyl-10-oxanthranol, aloe-emodin, rhein isomer	(Xu et al., 2018)
Aloe-emodin	SD rats	NA	Intestinal bacteria	Hydrolysis, hydroxylation, acetylation, demethylation	3-acetoxy–1,8-dihydroxy-6-hydroxymethyl-10-oxanthranol, 2-formyl-1,8-dihydroxy-3-hydroxymethyl-6-methoxyanthraquinone	(Song et al., 2011)
Aloe-emodin	*In vitro*	0.0156 mg/mL	Human intestinal bacteria	Reduction, methylation	O-methyl-aloe-emodin, 1,8-dihydroxy-3-hydroxymethyl-9-oxanthranol or 1,8-dihydroxy-3-hydro-xymethyl-10-oxanthranol and aloe-emodin isomer	(Huang et al., 2019)
Aloe-emodin 1/8-O-glycoside	*In vitro*	0.5 mL	Intestinal bacteria	Hydrolysis, reduction, substitution reaction	aloe-emodin, and reduction and acetoxyl derivatives	(Song et al., 2012)
Aloe-emodin-8-O-β-D-glycoside	SD rats	0.0240 mg/mL	Liver microsomes	Hydrolysis, hydroxylation, reduction, oxidation	aloe-emodin-8- O-β-D -glucopyranoside, aloe-emodin isomer, hydroxy-aloe-emodin, aloe-emodin, rhein	(Xu et al., 2018)
Aloe-emdion- *O*-glucopyranoside	*In vitro*	1 ml Xiao-Cheng-Qi Decoction solution (1g/ml raw formula herbs), including rhei Radix et Rhizoma (wine processed), Aurantii Immaturus Fructus and Magnoliae officinalis Cortex	Human intestinal bacteria	Hydrolysis and oxidation	aloe-emdion, rhein and rheinanthrone	(Liu et al., 2018b)
11-*O*-actyl-aloe-emdion-*O*-*β*-glc-xyl	*In vitro*	1 ml Xiao-Cheng-Qi Decoction solution (1g/ml raw formula herbs), including rhei Radix et Rhizoma (wine processed), Aurantii Immaturus Fructus and Magnoliae officinalis Cortex	Human intestinal bacteria	Hydrolysis and oxidation	aloe-emdion, rhein and then rheinanthrone	(Liu et al., 2018a)
Chrysophanol	SD rats	10 mL/kg rhubarb decoction	i.g.	Glucuronidation, sulfation	Chrysophanol-1-O-glucoside-8-O-glucuronide, chrysophanol-8-O-glucoside-1-O-glucuronide, chrysophanol-1,8-biglucuronides, chrysophanol-1-O-glucuronide, chrysophanol-8-O-glucuronide	(Song et al., 2010)
Chrysophanol	SD rats	0.0755 mg/mL	Liver microsomes	Hydroxylation, acetylation, demethylation, hydroxylation, reduction, oxidation	Chrysophanol, dihydroxy-chrysophanol, dihydroxyl-1,8-dihydroxy-3-methyl-9-oxanthranol/dihydroxyl-1,8-dihydroxy-3-methyl-10-oxanthranol, hydroxy-chrysophanol, rhein	(Xu et al., 2018)
Chrysophanol	SD rats	NA	Liver microsomes	Monohydroxylation, dihydroxylation	Chrysophanol, 1,4,8-trihydroxy-3-hydroxymethylanthraquinone, 2-hydroxychrysophanol, 4-hydroxychrysophanol	(Song et al., 2009a)
Chrysophanol	SD rats	NA	Intestinal bacteria	Hydrolysis, hydroxylation, acetylation, demethylation	3-acetoxy-1,8-dihydroxy-6-methyl-10-oxanthanol, 1,8-dihydroxy-2-(acetoxy) methyl-6-methylanthraquinone, 1,8-dihydroxy-2-(1-hydroxyethoxy) methyl-6-methylanthraquinone	(Song et al., 2011)
Chrysophanol	*In vitro*	0.0755 mg/mL	Human intestinal bacteria	Reduction, hydrolysis, acetylation, oxidation, demethylation, methylation, hydroxylation, dehydroxylation	Chrysophanol isomer, O-methyl-hydroxy-chrysophanol, aloe-emodin, O-methyl-chrysophanol, 1,8-dihydroxy-3-methyl-9-oxanthranol or 1,8-dihydroxy-3-methyl-10-oxanthranol, emodin, acetyl-1,8-di-hydroxy-anthraquinone, danthron, rhein	(Huang et al., 2019; Tian., et al., 2012)
Chrysophanol-1/8-O-glucoside	*In vitro*	0.5 mL	Intestinal bacteria	Hydrolysis, reduction, substitution reaction	Chrysophanol and then reduction and acetoxyl derivatives	(Song et al., 2012)
Chrysophanol-O-glucopyranoside	*In vitro*	1 ml Xiao-Cheng-Qi Decoction solution (1g/ml raw formula herbs), including rhei Radix et Rhizoma (wine processed), Aurantii Immaturus Fructus and Magnoliae officinalis Cortex	Human intestinal bacteria	Hydrolysis and oxidation	Chrysophanol, rhein and then rheinanthrone	(Liu et al., 2018a)
Emodin	Wistar rats	50 mg/kg	i.g.	Methylation, hydroxylation, oxidation	physcion, chrysophanol, aloe emodin, danthron, rhein	(Tian et al., 2012)
Emodin	SD rats	8 g/kg Zhi-Zi-Da-Huang decoction	i.g.	Glucuronidation, sulfation	Emodin-1-O-glucuronide, emodin-1-O-sulfate, emodin-3-O-glucuronide, emodin-3-O-sulfate	(Zhu et al., 2015)
Emodin	SD rats	2.26 mg/kg	i.g.	Oxidation, acidification, methylation, glucuronidation, sulfation	Emodin methylate, ω-hydroxy-emodin, 6-carboxyl emodin, physcion, emodin, sulfonyl emodin, emodin-di-glucuronide, emodin-glucuronide, emodin-glucuronide oxidate, emodin-sulfate oxidate	(Zhang et al., 2018b)
Emodin	SD rats	10 mL/kg rhubarb decoction	i.g.	Glucuronidation, sulfation, hydroxylation, hydrogenation, oxidation	emodin-O-diglucuronides, emodin-O-glucoside-O-glucuronide, 1,8-Dihydroxy-3-carboxy-6-methylanthraquinone-1or 8-O-glucoside, emodin-1 or 8-O-glucuronide-3-O-sulfate or emodin-1 or 8-O-sulfate-3-O-glucuronide, 1,3,8-trihydroxy-6-methyl-10-oxanthranol glucuronide, emodin-O-diglucuronides, 1,3,8-trihydroxy-6-(glucuronidyl)methylanthrquinone, emodin acid-O-glucuronide, emodin-2-C-glucuronide, emodin-3-O-glucuronide	(Song et al., 2010)
Emodin	SD rats	Raw root of P. multiflorum Thunb extract (10 mL/kg^/^, 2 g/mL)	i.g.	Glucuronidation, sulfation, oxidation	Emodin glucuronide sulfate, emodin 1, 8-O-diglucuronide, emodin 1, 3-O-diglucuronide, emodin 3, 8-O-diglucuronide, 4-hydroxyemodin, 5-hydroxyemodin, emodin acid-3-O-glucuronide, emodin acid-3-O-sulfate, physcion-glucuronides	(Huang et al., 2018)
Emodin	SD rats	0.0156 mg/mL	Liver microsomes	Transhydroxylation, hydroxylation, reduction, dehydroxylation, oxidation	Hydroxy-emodin, 1,3,8-trihydroxy-6-methyl-9-oxanthranol/1, 3,8-trihydroxy-6-methyl -10-oxanthranol, dihydroxy-emodin, hydroxy-emodin, aloe-emodin isomer, hydroxy-rhein, hydroxyl-aloe-emodin, aloe-emodin, emodin	(Xu et al., 2018)
Emodin	SD rats	NA	Liver microsomes	Hydroxylation	ω-hydroxyemodin, 2-hydroxyemodin, 4-hydroxyemodin, emodin acid, 3-carbomethoxy-6-methoxy-1,8-dihydroxyanthraquinone, physcion	(Song et al., 2008)
Emodin	SD rats	NA	Liver microsomes/intestinal bacteria	Monohydroxylation, methylation, oxidation in side chain	Emodin, physcion, 1, 3, 8-trihydroxy-6-(acetoxy) methyl-10-oxanthranol, ω-hydroxyemodin, 2-hydroxyemodin, 4-hydroxyemodin, emodin acid, 3-carbomethoxy-6-methoxy-1,8-dihydroxyanthraquinone, 1,8-dihydroxy-3-hydroxymethyl-10-oxanthranol	(Song et al., 2008; Song et al., 2009a; Song et al., 2011)
Emodin	*In vitro*	0.1950 mg/mL	Human intestinal bacteria	Acetylation, hydroxylation, methylation, trans hydroxylation, reduction	Aloe-emodin, isomer of emodin, 8-O-methyl-emodin, 1-O-methyl-emodin,3-O-methyl-emodin, 2-hydroxy-emodin, 4-hydroxy-emodin, ω-hydroxy-emodin, acetyl-1,3,8-trihydroxy-6-methyl-9-oxan-thranol or acetyl-1,3,8-trihydroxy-6-methyl-10-oxanthranol, acetyl-hydroxy-emodin	(Huang et al., 2019)
Emodin-1/8- O-glucoside	*In vitro*	0.5 mL	Intestinal bacteria	Hydrolysis, reduction, substitution reaction	Emodin and then reduction and acetoxyl derivatives	(Song et al., 2012)
Emodin-8-O-β-D-glucoside	SD rats	0.01 mg/mL	Liver microsomes	Transhydroxylation, hydrolysis, oxidation, hydroxylation	Dihydroxyl-1, 3, 8-trihydroxy-6-methyl-9-oxanthranol/dihydroxyl-1, 3, 8-trihydroxy-6-methyl-10-oxanthranol, hydroxy-emodin-O-glucopyranoside, hydroxy-emodin-O-glucopyranoside, emodin-8-O-β-glucopyranoside, emodin	(Xu et al., 2018)
Emodin-*O*-glucopyranoside	*In vitro*	1 ml Xiao-Cheng-Qi Decoction solution (1g/ml raw formula herbs), including rhei Radix et Rhizoma (wine processed), Aurantii Immaturus Fructus and Magnoliae officinalis Cortex	Human intestinal bacteria	Hydrolysis and oxidation	Emodin, rhein and then rheinanthrone	(Liu et al., 2018a)
Physcion	SD rats	NA	i.g.	Glucuronidation, sulfation	Physcion oxidate, physcion-sulfate, physcion-glucuronide	(Zhang et al., 2018a)
Physcion	SD rats	10 mL/kg rhubarb decoction	i.g.	Glucuronidation, sulfation	Physcion-1-O-glucoside-8-O-glucuronide or physcion-8-O-glucoside-1-O-glucuronide, physcion-1, 8-O-diglucuronides	(Song et al., 2010)
Physcion	SD rats	NA	Liver microsomes	Monohydroxylation, oxidation in side chain, demethylation	Emodin, 1,8-dihydroxy-3-methoxyanthraquinone, 1,8-dihydroxy-3-hydroxymethyl-6-methoxyanthraquinone, hydroxyphyscion, emodin acid, ω-hydroxyemodin, 4-hydroxyemodin, 3-carbomethoxy-6-methoxy-1,8-dihydroxyanthraquinone	(Song et al., 2009a)
Physcion	SD rats	0.16 mg/mL	Liver microsomes	Demethylation, hydroxylation, reduction	Dihydroxy-1,8-dihydroxy-3-methoxy-6-methyl-9-oxanthranol/1, 8-dihydroxy-3-methoxy-6- methyl-10-oxanthranol, emodinIsomer, hydroxy-emodin, emodin, physcion	(Xu et al., 2018)
Physcion	SD rats	NA	Intestinal bacteria	Hydrolysis, hydroxylation, acetylation, demethylation	2-Formyl-1,8-dihydroxy-3-hydroxymethyl-6-methoxyanthraquinone, 1,8-dihydroxy-2-(acetoxy) methyl-3-methoxyanthraquinone, 3-acetoxy -1,8-dihydroxy-6-(acetyl) methylanthraquinone	(Song et al., 2011)
Physcion	*In vitro*	0.1610 mg/mL	Human intestinal bacteria	demethylation, dehydroxylation, transhydroxylation	Chrysophanol isomer, physcion isomer, aloe-emodin, emodin	(Huang et al., 2019)
Physcion-O-glucoside	*In vitro*	0.5 mL	Intestinal bacteria	Hydrolysis, reduction, substitution reaction	physcion and then reduction and acetoxyl derivatives	(Song et al., 2012)
Rhein	SD rats	8 g/kg Zhi-Zi-Da-Huang decoction	i.g.	glucuronidation, sulfation	Rhein-1-O-sulfate, rhein-8-O-sulfate, rhein-8-O-glucuronide, rhein-1-O-glucuronide	(Zhu et al., 2015)
Rhein	SD rats	10 mL/kg rhubarb decoction	i.g.	glucuronidation, sulfation	rhein, rhein-1-O-glucoside	(Song et al., 2010)
Rhein	SD rats	NA	Liver microsomes	Hydrogenation, methylation	1,8-dihydroxy-3-carboxy-9-oxanthranol, 1,8-dihydroxy-3-carboxy-10-oxanthranol, 2-methylrhein	(Song et al., 2009a)
Rhein	SD rats	0.1950 mg/mL	Liver microsomes	Hydroxylation, reduction	rhein, rhein isomer, dihydroxyl-1,8-dihydroxy-3-carboxyl-9-oxanthranol/dihydroxyl-1,8-dihydroxy-3-carboxyl-10-oxanthranol	(Xu et al., 2018)
Rhein	SD rats	NA	Intestinal bacteria	Hydrolysis, hydroxylation, acetylation, demethylation	2-acetoxy -6-carboxy -1,8-dihydroxyanthraquinone, 3-acetoxy–1,8-dihydroxy-6-hydroxymethyl-10-oxanthranol	(Song et al., 2011)
Rhein	*In vitro*	0.0350 mg/mL	Human intestinal bacteria	methylation, hydroxylation, reduction	rhein, O-methyl-rhein, 1,8-dihydroxy-3-carboxyl-9-oxanthranol, 1,8-dihydroxy-3-carboxyl-10-oxanthranol, hydroxy-rhein, chrysophanol isomer	(Huang et al., 2019)
Rhein	*In vitro*	1 ml Xiao-Cheng-Qi Decoction solution (1g/ml raw formula herbs), including rhei Radix et Rhizoma (wine processed), Aurantii Immaturus Fructus and Magnoliae officinalis Cortex	Human intestinal bacteria	Hydrolysis	Rheinanthrone	(Liu et al., 2018a)
Rhein-8-O-glucoside	SD rats	0.025 mg/mL	Liver microsomes	Hydrolysis, hydroxylation, reduction	Rhein-8-O-glucopyranoside, dihydroxy-3-carboxyl-9-oxanthranol-O-glucopyranoside/1, 8-dihydroxy-3-carboxyl-10-oxanthranol-O-glucopyranoside, rhein, emodin isomer	(Xu et al., 2018)
Sennoside A	Human	0.0250 mg/mL	Intestinal bacteria	Hydrolysis, methylation, hydroxylation, dehydroxylation, reduction	sennidine A-8-O-monoglucoside, rheinanthrone, dehydroxy-rheinanthrone, O-methyl-hydroxy-rheinanthrone, rhein	(Huang et al., 2019)
sennoside A	*In vitro*	1 ml Xiao-Cheng-Qi Decoction solution (1g/ml raw formula herbs), including rhei Radix et Rhizoma (wine processed), Aurantii Immaturus Fructus and Magnoliae officinalis Cortex	Human intestinal bacteria	Hydrolysis	Rheinanthrone	(Liu et al., 2018a)
Sennoside B	Human	0.0393 mg/mL	Intestinal bacteria	Hydrolysis, methylation, hydroxylation, dehydroxylation, reduction	Sennoside A, dehydroxy-rheinanthrone, O-methyl-rheinanthrone, sennidine B-8-O-monoglucoside, sennidine A-8-O-monoglucoside, aloe-emodin, O-methyl-hydroxy-rheinanthrone, O-methyl-rheinanthrone, rhein	(Huang et al., 2019)
Sennoside C	Human	0.0398 mg/mL	Intestinal bacteria	Hydrolysis, oxidation, methylation, dehydroxylation, reduction	sennoside C, sennidine C-8-monoglucoside, sennidine C-8′-monoglucoside, rheinanthrone-8-O-monoglucoside, dehydroxy-rheinanthrone, rhein, aloe-emodin, O-methyl- rheinanthrone	(Huang et al., 2019)
Sennoside D	Human	0.0263 mg/mL	Intestinal bacteria	Hydrolysis, oxidation, methylation, dehydroxylation, reduction	Chrysophanol isomer, sennidine D-8-O-monoglucoside or sennidine D-8′-O-monoglucoside, O-methyl-rheinanthrone, aloe-emodin, rhein	(Huang et al., 2019)

NA: not available; i. g.: intragastrical administration; I.V.: intravenous injection.

### Hydrolysis

Anthraquinone glycosides can be hydrolyzed by both intestinal bacteria and liver enzymes. Song et al. incubated processed rhubarb aqueous extracts with rat intestinal bacteria and found that 12 anthraquinone glycosides were hydrolyzed into anthraquinone aglycones, aloe-emodin, chrysophanol, emodin, and physcion respectively (Song et al., 2012) (Table 4). For anthraquinone glycoside-containing formulae, Liu and colleagues incubated Xiao-Cheng-Qi decoction (XCQD) with human intestinal bacteria *in vitro* and found that sennoside A and seven other anthraquinone glycosides were hydrolyzed (Liu X. Y. et al., 2018). It is worth noting that anthraquinone glycosides, such as aloe-emodin-8-O-β-D-glucopyranoside, emodin-8-O-β-D-glucopyranoside, and rhein-8-O-β-D glucopyranoside can also be transformed into their aglycones by the enzymes in the liver (Xu et al., 2018).

### Glucuronidation

Glucuronidation in the intestines and liver is one of the main phase II metabolic reactions of anthraquinones. UGTs play a pivotal role in the glucuronidation of anthraquinones (Wu et al., 2014; Meng and Ding, 2019). When oral administered with Zhi-Zi-Da-Huang decoction (ZZDHD), which consists of *Gardenia jasminoides* Ellis (Zhizi), *Rheum palmatum* L. (Dahuang), *Citrus aurantium* L. (Zhishi) and *Sojae Semen* Praeparatum (Dandouchi), emodin and rhein can be transformed to rhein-8-O-glucuronide, rhein-1-O-glucuronide, emodin-1-O-glucuronide, and emodin-3-O-glucuronide (Zhu et al., 2015). Aloe-emodin is transformed to glucuronidation forms by β-glucuronidase and sulfatase/β-glucuronidase following intravenous and oral administration in rats (Yu et al., 2016).

Da-Huang-Xiao-Shi decoction (DHXSD) is another formula for treating jaundice. It is composed of four crude drugs: *Rheum officinale* Baill (Dahuang), *Gardenia jasminoides* Ellis (Zhizi), *Phellodendron amurense* Rupr. (Huangbo), and Natrii Sulfas. When DHXSD was orally administered to rats, six anthraquinone glucuronidation, aloeemodin-O-glucuronide, chrysophanol-O-glucoside-O-glucuronide, rhein-O-glucuronide, physcion-O-glucoside-O-glucuronide, chrysophanol-O-glucuronide, and emodin-O-glucuronide were transformed to glucuronidation forms (Wang D. et al., 2017) (Table 4).

### Sulfonation

Sulfonation in the intestines and liver is the other main phase II metabolic reaction of anthraquinones by sulfotransferase (SULT) (Song, et al., 2010). Like glucuronidation, the sulfonation is another detoxification process. Additionally, sulfonated anthraquinones can be used as a remedy strategy for free radical-related diseases such as AAPH (2,2′-azobis (2-amidinopropane hydrochloride))-induced hemolysis (Shia et al., 2009; Shia et al., 2010).

Aloe-emodin, chrysophanol, emodin, physcion, and rhein are metabolized to sulfonation forms (Song, et al., 2010; Zhu et al., 2015; Zhang, et al., 2018a; Huang et al., 2018). This can lead to a decline in the oral bioavailability of anthraquinones (Teng et al., 2007; Shia et al., 2009) (Table 4).

### Methylation/Demethylation

Methylation is another metabolic reaction for anthraquinones in both the intestines and the liver (Song Z. et al., 2009). Aloe-emodin (Song R. et al., 2009), chrysophanol (Huang et al., 2019), emodin (Tian et al., 2012), rhein (Song R. et al., 2009; Huang et al., 2019) and rheinanthrone (Huang et al., 2019) are methylated to O-methyl-aloe-emodin, O-methyl-chrysophanol, 8-O-methyl-emodin, O-methyl-rhein, and O-methyl-rheinanthrone, respectively. O-methyltransferase may be involved in the methylation process (Koyama et al., 2009; Huang et al., 2019). Conversely, demethylation is an opposite reaction in anthraquinone metabolic processes. The demethylation of chrysophanol is transformed to dihydroxy-chrysophanol, while physcion is transformed to emodin/isomer (Xu et al., 2018). Of note, the rapid demethylation of physcion to emodin may be the reason why the bioavailability of physcion is low (Song R. et al., 2009).

### Hydroxylation/Dehydroxylation

The hydroxylation of emodin is hydroxy-emodin and dihydroxy-emodin. Chrysophanol can also be transformed to hydroxylation forms as hydroxy-chrysophanol and dihydroxy-chrysophanol (Xu et al., 2018). Hydroxylation is also the synthesis pathway to form anthraquinone glycosides. Additionally, aleo-emodin is transformed to aloe-emodin-8-O-glucoside-1-O-glucuronide or aloe-emodin-1-O-glucoside-8-O-glucuronide, 2-hydroxyaloe-emodin-ω-O-glucuronide through hydroxylation, glucuronidation, hydrogenation, and oxidation (Song et al., 2010). Cytochromosome P450s, including CYP1A2, CYP2C19, CYP2B6, and CYP3A4, play major roles in the hydroxylation of anthraquinones (He et al., 2015; Qin et al., 2018). In contrast, emodin (Xu et al., 2018) and rheinanthrone (Huang et al., 2019) are dehydroxylated to chrysophanol isomers, and dehydroxy-rheinanthrone, respectively. Other hydroxylation and dehydroxylation are listed in Table 4.

### Oxidation/Reduction (Hydrogenation)

For oxidation, chrysophanol (Xu et al., 2018), emodin (Zhang J. et al., 2018), physcion (Song R. et al., 2009), rheinanthrone (Huang et al., 2019) and aloe-emodin anthrone (Huang et al., 2019) are oxidized to ω-hydroxy-emodin, rhein and aloe-emodin in the intestines and liver. Aloe-emodin (Song R. et al., 2009; Song et al., 2010; Xu et al., 2018) is oxidized to rhein. The oxidation reaction can decrease the bioavailability of anthraquinones. The order of bioavailability of some anthraquinones is: rhein > emodin > chrysophanol > aloe-emodin. This may result from that sennosides A and B, aloe-emodin and chrysophanol all being oxidized to rhein (Shia et al., 2011a). CYP1A2, CYP2B6 and CYP3A4 are the major enzymes for oxidation (Sun et al., 2018).

For reduction, aloe-emodin, chrysophanol, emodin, physcion, rhein and rhein-8-O-glycopyranoside are hydrogenated (Xu et al., 2018; Yu et al., 2018; Huang et al., 2019). (Table 4).

### Acetylation

Chrysophanol, emodin, physcion and rhein can be acetylated into acetyl-1,8-dihydroxy-anthraquinone, acetyl-1,3,8-trihydroxy-6-methyl-9-oxanthranol and 1,8-dihydroxy-2-(acetoxy) methyl-3-methoxyanthraquinone, respectively (Song et al., 2011; Xu et al., 2018; Huang et al., 2019).

### Esterification

Rhein is Esterified to Rhein Methyl Ester by intestinal Flora (Fan et al., 2016).

### Affecting Factors

#### Physiological Condition

*Sex* The glucuronidation of emodin shares the same rate in human males and females, while the rates in females are faster than the rates in male rats, guinea pigs, and dogs. However, at an emodin concentration of 2.5 μM, male mice have a higher rate of glucuronidation than females (Liu et al., 2010). In addition, danthron and chrysophanol produced from emodin metabolism are only present in male rats (Tian et al., 2012). The bioavailability of rhein in female rats is higher than that in males. The mechanism may be the different activation of UGTs between the male and female (Zhang et al., 2015)**.**

#### Disorders

The glucuronidation and hydrolysis of anthraquinones and their glycosides are reduced in rats with ulcerative colitis. The mechanism may be that colitis reduces the activities of β-glucosidases and β-glucuronidases in the intestinal flora (Wu W. J. et al., 2017). In alcohol-induced liver injury, the metabolism of aloe-emodin, chrysophanol, physcion, aurantio-obtusin, chrysoobtusin, emodin, obtusin and rhein increase. This may result from that alcohol induces P450 (e.g., CTP2E1, CYP3A and CYP1A) (Shao and Feng, 2015; Li P. et al., 2017). Furthermore, the metabolism of rhein decreases under acute liver injury because of the lower expression and activity of CYP450, especially in males (Zhang et al., 2015).

#### Drugs

*Drug–Drug Interactions* Preparations with wine are very common for Chinese medicines. Thus the role of wine (ethanol) in Chinese medicines has attracted more research interest. Studies have shown that Rhei Radix et Rhizoma steamed with wine can accelerate the hydrolysis of anthraquinone glycosides in rats. This results in higher bioavailability of emodin, physcion and chrysophanol (Zhang et al., 2019). Additionally, wine reduces the T_1/2_ of aloe-emodin and emodin in Rhei Radix et Rhizoma (Wu Y. et al., 2017). This may be consistent with the traditional Chinese medicine theory of drug processing (known as Paozhi): wine promotes blood circulation. It is very common for ethanol to be used for drug processing of Chinese medicine to induce bioavailability, enhance efficacy and/or decrease adverse drug reactions.

For anthraquinone compounds, piperine increases the AUC and *C*_max_ of emodin by inhibiting UGTs (Di et al., 2015). Synergism can also occur between different anthraquinones. Sennoside A is an active anthraquinone glucoside in rhubarb (Rhei Radix et Rhizoma) for treating constipation. Rhein 8-O-β-D-glucopyranoside, emodin, aloe-emodin and rhein can enhance the purgative action of sennoside A by accelerating its hydrolysis by inducing intestinal bacteria (Takayama et al., 2012).

Furthermore, the different classes of compounds in the same herb may influence the PKs of anthraquinones. 2,3,5,4-Tetrahydroxy-stilbene-2-O-β-D-glycoside (TSG), a compound in Polygini Multiflori Radix (Heshouwu) (Li et al., 2016; Li H. et al., 2017) inhibits the mRNA expression of the UGT isoforms, UGT1A8, UGT1A10, and UGT12B7, leading to a decrease in glucuronidation of emodin (Ma et al., 2013; Yu et al., 2017). Inhibiting emodin glucuronidation will increase the bioavailability of emodin; however, it also leads to an accumulation of emodin to induce liver damage (Ma et al., 2015). Interestingly, TSG also accelerates metabolism to clear emodin by enhancing the activity of CYP1A2 (Xing et al., 2019), indicating that the interaction role of TSG in emodin pharmacological and toxicological actions is complex and needs to be further studied.

Rhei Radix et Rhizoma exerts purgative action for constipation. However, hepatotoxicity and abdominal pain limit its clinical application. When using Rhei Radix et Rhizoma combination with Glycyrrhizae Radix et Rhizoma (Gancao) (Da-Huang-Gancao Decoction in Chinese, Daiokanzoto in Japanese), hepatotoxicity and abdominal pain were reduced. The underlying mechanisms may be due to Glycyrrhizae Radix et Rhizoma inducing P450 to accelerate the transformation of emodin (Han et al., 2010). Furthermore, liquiritin and liquiritin apioside in Glycyrrhizae Radix et Rhizoma can induce intestinal bacteria to intensify the metabolism of sennoside A and enhance purgative action (Matsui et al., 2011). Increasing research on the intestinal flora may provide more insights into the novel role of intestinal bacteria in the PKs of anthraquinones.

Dahuang Fuzi decoction is the combination of Rhei Radix et Rhizoma, Aconiti Lateralis Radix Praeparata (Fuzi) and Asari Radix et Rhizoma (Xixin). Drug extrusion by intestinal P-gp can both reduce drug absorption and modulate the effects of inhibitors and inducers of CYP3A/CYP3A4-mediated metabolism. The study has shown that the compounds from Aconiti Lateralis Radix Praeparata or Asari Radix et Rhizoma may induce P-gp and CYP3A/CYP3A4, leading to a decrease in AUC and *C*_max_ for anthraquinones (Liu et al., 2015).

Xin et al. reported that San-Huang-Xie-Xin decoction (SHXXD), including Rhei Radix et Rhizoma, Scutellariae Radix and Coptidis Rhizoma (containing berberine), showed increases in the *C*_max_ and AUC of rhein compared with the single herb Rhei Radix et Rhizoma (Xin et al., 2009). The mechanisms may be due to the inhibited glucuronidation activity of UGTs for rhein by other ingredients in SHXXD (Hou et al., 2014).

The metabolic pathways and metabolites of anthraquinones are listed in Table 4.

## Excretion

### Excretion Routes and Form

Generally, anthraquinones are mainly excreted via the kidney (Chen et al., 2014), recta (Zhang M. et al., 2018), and/or gallbladder (Ma et al., 2005) via prototypes and/or metabolites. They are excreted with urine (Ma et al., 2005), feces (Zhang J. et al., 2018), and/or bile (Ma et al., 2005).

Anthraquinones excreted through bile may be reabsorbed and utilized in the intestines to form a hepatointestinal circulation, so they can be excreted for a long time (Yang B. et al., 2019). The amount of chrysophanol excreted through urine is significantly greater than that excreted through bile (Ma et al., 2005). The urinary excretion of emodin is 1.5-folds that of feces (Sun et al., 1986; Wu et al., 2008; Du et al., 2014). Regarding metabolite elimination of anthraquinones, e.g., rhein, the metabolite of emodin, exists in the plasma for a short time because of the rapid excretion (Tian et al., 2012).

Glucuronic acid and sulfuric acid conjugates of rhein are dominant in urine and fecal excreta. Only 20% of the prototype rhein is excreted in urine and feces (Wan et al., 2013).

### Affecting Factors

#### Physiological Condition

*Species* Physcion can be detectable in the urine of humans rather than in that of rats. However, there is an opposite result for rhein between humans and rats. In addition to differences in dosage and detection instruments, this species diversity may result from apparent distribution volume (Li et al., 2003).

*Sex* The excretion of danthron and rhein in male rats is faster than that in female (Tian et al., 2012). The excretion of emodin glucuronide is slower in male rats than that in female rats (Liu W. et al., 2011).

*Food* Feeding increases the half times of elimination (*T*_1/2_) of emodin and rhein, possibly because feeding stimulates an increase in bile secretion to form hepato-intestinal circulation. Additionally, feeding inhibits the activity and the saturation of the related metabolic enzymes and consequently increases the T_1/2_ of emodin and rhein (Gong et al., 2011).

#### Disorders

The mean residence times (MRTs) of anthraquinones, e.g., aloe-emodin, chrysophanol, emodin, physcion, and rhein are prolonged in microcirculation disorder (Dai et al., 2014; Yan and Dai, 2014; Zhu et al., 2017). For ischemic cerebrovascular disease, the elimination s of aloe-emodin, emodin, and rhein are significantly decreased in thrombotic cerebral ischemia compared with normal condition in rats (Feng et al., 2013). The T_1/2_ values of chrysophanol and rhein are increased in acute pancreatitis, and the plasma clearance rates (CL) are decreased (Gong et al., 2009; Yang et al., 2012). Regarding liver disorders, the MRT of rhein is shortened and elimination is accelerated in acute liver injury rats (Zhang et al., 2015)**.** However, in the other reports, the T_1/2_ values of aloe-emodin, chrysophanol, emodin and rhein increase (Li P. et al., 2017; Yang N. et al., 2019). The contradict results may result from the different animal models. For alcoholic liver injury, the T_1/2_ and MRT of emodin in rats are prolonged, and CL is decreased (Zhu et al., 2016). In addition, studies have reported that gastrointestinal disorders caused by alcoholic liver injury may affect the excretion of drugs (Burkard et al., 2005; Luo et al., 2014). The T_1/2_ of chrysophanol and rhein increases in rats with ulcerative colitis (Wu W. J. et al., 2017). Under chronic renal failure conditions, the elimination of rhein is accelerated in rats due to urine alkalization and an increase in urine output (Wang et al., 2009).

The T_1/2_ values of chrysophanol and rhein in Rhei Radix et Rhizoma are increased in lipopolysacchoride (LPS)-induced inflammation. However, the underlying mechanisms are unkown (Li et al., 2013c).

#### Drugs

*Drug–Drug Interactions* For drug compatibility, combination with Scutellariae Radix increases the urinary excretion of emodin in Rhei Radix et Rhizoma compared with oral administration of Rhei Radix et Rhizoma alone in rats (Wu et al., 2010; Li J. et al., 2018). Glycyrrhizae Radix et Rhizoma increases the elimination rate of rhein in Rhei Radix et Rhizoma. This may attenuate the hepatotoxicity of rhein in Rhei Radix et Rhizoma (Han et al., 2010).

The compatibility of Rhei Radix et Rhizoma and Aconiti Lateralis Radix Praeparata (Fuzi) is the basic herb pair applied in many traditional Chinese prescriptions. Studies have shown that Aconiti Lateralis Radix Praeparata decreases the clearance of aloe-emodin, chrysophanol and rhein. Therefore, the safety of the herb pair Rhei Radix et Rhizoma and Aconiti Lateralis Radix Praeparata should be given more attention (Li et al., 2015).

For the formula Dahuang-mudan decoction (DMD), in which Rhei Radix et Rhizoma is combined with Magnoliae Officinalis Cortex, Aurantii Fructus Immaturus, and Natrii Sulfas, Zhang reported that the prolonged elimination of aloe-emodin and emodin, indicating a lower toxicity in this formula. The underlying mechanisms may be due to competitive inhibition between the chemical compounds in DMD and need to be further investigated (Nong et al., 2019). An eight-herb formula Niu-Huang-Jie-Du tablets (NHJDT), including Bovis Calculus (Niuhuang), Rhei Radix et Rhizoma, Realgar (As_2_S_2_, Xionghuang), Gypsum Fibrosum (CaSO_4_·2H_2_O, Shigao), Platycodonis Radix (Jiegeng), and Borneolum Syntheticum (D-borneoland, Bingpian), exerts heat-clearance and detoxicification in Chinese medicine. The data showed that the clearance of chrysophanol isomers in NHJDT increased in rats, indicating that drug-drug interaction for excretion occured between the ingredients in NHJDT. However, the mechanism is still unknown (Liu Y. et al., 2018).

The elimination of anthraquinones is listed in Table 5.

**TABLE 5:** The elimination of anthraquinones.

Pharmacokinetic parameters	T_1/2_ (h)		CL L/Kg·h		References
	Rats	Dogs	Rats	Dogs	
Aloe-emodin	0.27–162.12	2.02–14.73	0.002–166.76	61.63	(Feng et al., 2012; Yang et al., 2012; Li et al., 2013b: Zhang et al., 2013a; Feng et al., 2014;
Alizarin	8.97	–	–	–	(Gao et al., 2018)
Aurantio-obtusin	4.94–13.78	–	1.88	–	(Zhang et al., 2014; Yang et al., 2015; Yang et al., 2019a)
Chrysophanol	0.36–20.99	1.95–15.18	0.001–44.74	146.61	(Yang et al., 2012; Feng et al., 2013; Feng et al., 2014; Jiang et al., 2015; Zhu et al., 2017)
Chrysophanol-8-O-β-d-glycoside	4.8	–	–	–	(Ullah et al., 2018)
Chryso-obtusin	3.86–8.69	–	3.04	–	(Zhang et al., 2014; Yang et al., 2019a)
Citreorosein	3.97	–	–	–	(Cheng et al., 2020)
Emodin	0.10–53.99	1.72–18.73	0.006–56.4	17.12	(Song et al., 2009a:; Yang et al., 2012; Li et al., 2013b; Zhang et al., 2013a; Zhang et al., 2013b; Feng et al., 2014; Zhu et al., 2014; Zhang et al., 2018c)
Emodin-8-O-β-D-glycoside	0.18–3.92	–	–	–	(Zhang et al., 2018b; Cheng et al., 2020)
Munjistin	9.22–11.97	–	–	–	(Gao et al., 2016; Gao et al., 2018)
Obtusifolin	1.87–11.12	–	21.10	–	(Zhang et al., 2012; Yang et al., 2015; Yang et al., 2019a)
Obtusin	4.41–8.28	–	1.96	–	(Zhang et al., 2014; Yang et al., 2019a)
Physcion	0.28–39.12	13.08	10.10–27.35	109.53	(Feng et al., 2013; Feng et al., 2014; Feng et al., 2017; Zhu et al., 2017)
Physcion-8-O-β-D-glycoside	6.13–6.20	–	–	–	(Ullah et al., 2018; Cheng et al., 2020)
Purpurin	8.07–9.52	–	–	–	(Gao et al., 2016; Gao et al., 2018)
Questinol	8.90	–	–	–	(Cheng et al., 2020)
Rhein	0.15–39.39	1.8–10.11	0.002–17.2	0.98	(Yang et al., 2012; Zhang et al., 2013a; Li et al., 2013b; Zhang et al., 2013b; Feng et al., 2014; Zhu et al., 2014; Zhu et al., 2017; Zhang et al., 2018c)
Xanthopurpurin	8.1	–	–	–	(Han et al., 2020)
1-desmethylobtusin	7.01	–	1.33	–	(Zhang et al., 2014)

T_1/2_: half time of elimination; CL: plasma clearance rate.

## Discussion

Anthraquinones are naturally present in medicinal plants, especially Chinese medicines. They have attracted increasing research attention because of their pharmacological and toxicological effects. Thus, the approach to determining their PK plays a key role in exploring their actions and mechanisms. In this study, 33 out of 217 free anthraquinones and glycosides were studied for their PK (Tables 1–5 and Supplementary Table S1; Figure 1). This may result from well-investigated actions and/or detectable concentrations either in plants or *in vivo* for the 33 compounds. The other compounds without PK studies may be difficult to isolate from natural plants, undetectable and/or weak bioactions.

Regarding the factors influencing the PK of anthraquinones, it is suggested to consider all *in vivo* processes instead of absorption, distribution, metabolism or elimination alone. For example, there are multiple factors influencing the bioavailability of rhein. The differences *T*_max_ and AUC difference of rhein between females and males always invole complex factors, including different body weights, apparent distribution volumes and fat ratios (which are associated with absorption and distribution), phase Ⅰ and phase Ⅱ metabolism (other anthraquinone glycosides, sennoside A/B, aloe-emodin, can all be transformed into rhein and subsequently form a blood accumulation of rhein when multiple anthraquinone-containing medicinal herbs are administered) (Shia et al., 2011a; Zhang et al., 2015), and live and kidney blood flow and glomerular filtration rates (which link with the process of elimination) (Zhu et al., 2006).

In addition, with the increasing use of Chinese medicines, drug-drug interactions for anthraquinones in Chinese formulae affect all processes of PK. Even in a single herb, e.g., Polygoni Multiflori Radix (Heshouwu) (Figures 1C,F), the drug-drug interactions between the components are complicated. On the one hand, TSG inhibits UGTs and decreases the elimination of emodin to enhance the effects and toxicity of emodin (Ma et al., 2013; Yu et al., 2017). On the other hand, TSG induces the activity of CYPs and accelerates the elimination of emodin (Xing et al., 2019), which may attenuate the effects or toxicity of emodin. Our previous studies found that the anticancer efficacy of 400 μg/mL of ethanol extract of Polygoni Multiflori Radix (containing approximately 1.48 μM of emodin) (Li H. et al., 2018) was similar to that of 100 μM emodin alone (Yang N. et al., 2019). Given the different anticancer effects of anthraquinones (Yang et al., 2018), it is strongly suggested that there would be drug interactions between ingredients in Polygoni Multiflori Radix *in vivo*. Actually, they are transformed each other *in vivo* via intestinal flora, and/or liver enzymes (Li P. et al., 2017; Xu et al., 2018; Huang et al., 2019). This may increase their efficacy and/or toxicity. Therefore, it would be very important to rationally investigate the *in vivo* processes of anthraquinone-containing Chinese medicines in clinical settings.

Traditoinel Chiense medicine theory facilitates preparation and formulae using drug interactions for rational drug use. These methods are very commonly used for drug processing of Chinese medicine (known as Paozhi) to induce bioavailability, enhance efficacy and/or decrease adverse drug reactions. For example, ethanol can accelerate metabolism including hydrolysis of anthraquinones glycosides in Rhei Radix et Rhizoma. Thus emodin, physcion and chrysophanol have higher bioavailability in Rhei Radix et Rhizoma steamed with wine (Zhang et al., 2019). Another interesting example of drug interactions is the ancient classic formula Rhubarb Peony decoction (Da Huang Mu dan Tang) from the Han Dynasty of China. The formula consists of five components, Rhei Radix et Rhizoma, Moutan Radix Cortex, Persicae Semen, Benincasae Semen (Dongguazi) and Natrii Sulfas, among which Natrii Sulfas can decrease the *C*_max_ of rhein during absorption and metabolism. This results in the diminished toxicity of rhubarb in Rhubarb Peony decoction (Zhang Y. X. et al., 2013).

It is worth noting that the metabolism of anthraquinones extends to multiple processes and is transformed into multiple products. For example, processed rhubarb aqueous extracts with rat intestinal bacteria lead to the hydrolysis of 12 anthraquinone glycosides to anthraquinone aglycones. Then, the latter are subsequently transformed to reduction and acetoxyl derivatives (Song et al., 2012). For the anthraquinone glycoside-containing formula, Xiao-Cheng-Qi decoction (XCQD) incubated with human intestinal bacteria *in vitro* leads to the hydrolysis of six anthraquinone glycosides to aglycones. The latter are transformed to rhein, which is further hydrolyzed to rheinanthrones (Liu X. Y. et al., 2018) (Figure 1 and Table 4).

The PK of anthraquinones may be illustrated in Figure 1.

## Conclusion

Some anthraquinones and their glycosides, such as aloe-emodin, chrysophanol, emodin, physcion, rhein and sennosides, have attracted the most PK research interest due to their greater biological activities and/or detectability. Anthraquinones are mainly absorbed in the intestines and are mostly distributed in blood flow-rich tissues and organs. They may have two absorption peaks because of the hepato-intestinal circle, reabsorption in organs/tissues and glycoside hydrolysis. Drug-drug interactions influencing PK may provide insights into drug compatibility theory to enhance or reduce pharmacological/toxicological effects in Chinese medicine formulae and deserve deep investigation.

## Data Availability

The datasets supporting the conclusions of this article are included within the article and its additional files.
