# Supplementary material for: Pharmacokinetics of Anthraquinones from Medicinal Plants
Source: Front Pharmacol. 2021 Apr 15;12:638993. doi: 10.3389/fphar.2021.638993 (PMC8082241; doi:10.3389/fphar.2021.638993)
Supplement: Supplementary file 1 [file table1.docx]

Supplementary Table S1: The names and classification of anthraquinones

| Anthraquinones | CAS No. | References |
| --- | --- | --- |
| **Mononuclear anthraquinones** |  |  |
| 1, 2-dihydroxy-3-methyl anthraquinone | 602-63-1 | (Wu et al., 2009) |
| 1, 3-dihydroxy-2-methoxy anthraquinone | 10383-63-8 | (Yang et al., 2019b) |
| 1, 3-dihydroxy-6-methoxy-7-methyl anthraquinone | 255845-73-9 | (Chen et al., 2003) |
| 1,2,3,4-tetrahydro-3a-hydroxy-5,6-dimethoxy-2b-methyl anthraquinone (prisconnatanone B) | 1349680-87-0 | (Feng et al., 2011) |
| 1,2,3,4-tetrahydro-3a,5-dihydroxy-6,7-dimethoxy-2b-methyl anthraquinone (prisconnatanone A) | 1349680-84-7 | (Feng et al., 2011) |
| 1,2,3-trimethoxy-7-methyl anthraquinone | 1096158-40-5 | (Feng et al., 2011) |
| 1,2,4,6-tetrahydroxy anthraquinone | 75313-07-4 | (Son et al., 2007) |
| 1,2,7-trimethoxyl-6,8-dihydroxy-3-methylanthraquinone | 1622982-59-5 | (Guo et al., 2017) |
| 1,2-dimethoxy-6-methyl anthraquinone | 1268477-69-5 | (Li and Jiang, 2018) |
| 1,2-dimethoxy-8-hydroxy-3-methyl anthraquinone | 82868-99-3 | (Jia et al., 2009) |
| 1,3,6-trihydroxy-2-methyl anthraquinone | 87686-86-0 | (Itokawa et al., 1993) |
| 1,3,6-trihydroxy-2-methylanthraquinone-3-O-(3’ -O-acetyl)-α-rhamnosyl-(1→2)-β-glycoside | NA | (Itokawa et al., 1989) |
| 1,3,6-trihydroxy-2-methylanthraquinone-3-O-(3’,6’-O-diacetyl)-α-rhamnosyl-(1→2)-β-glycoside | NA | (Itokawa et al., 1989) |
| 1,3,6-trihydroxy-2-methylanthraquinone-3-O-(4’,6’-O-diacetyl)-α-rhamnosyl-(1→2)-β-glycoside | NA | (Itokawa et al., 1989) |
| 1,3,6-trihydroxy-2-methylanthraquinone-3-O-(6’ -O-acetyl)-α-rhamnosyl-(1→2)-β-glycoside | NA | (Itokawa et al., 1989) |
| 1,3,6-trihydroxy-2-methylanthraquinone-3-O-(6’ -O-acetyl)-α-xylopyranosyl-(1→2)-β-glycoside | NA | (Wang et al., 1992) |
| 1,3,6-trihydroxy-2-methylanthraquinone-3-O-(6’ -O-acetyl)-β-D-glycoside | NA | (Qiao et al., 1990) |
| 1,3,6-trihydroxy-2-methylanthraquinone-3-O-(6’ -O-acetyl)-β-D-xylose-(1→2)-β-D-glycoside | NA | (Wang et al., 1992) |
| 1,3,6-trihydroxy-2-methylanthraquinone-3-O-α-L-rhamnosyl-(1→2)-β-D-glycoside | NA | (Itokawa et al., 1989) |
| 1,3,6-trihydroxy-2-methylanthraquinone-3-O-α-rhamnosyl-(1→2)-β-glycoside | NA | (Itokawa et al., 1989) |
| 1,3,6-trihydroxy-2-methyl-anthraquinone-3-O-β-glycoside | 125906-49-2 | (Wang et al., 1992) |
| 1,3-dihydroxy-2-carbomethoxy anthraquinone | 79820-23-8 | (Mishchenko et al., 2007) |
| 1,3-dihydroxy-2-ethoxymethyl anthraquinone (lucidin ethyl ether/ ibericin) | 17526-17-9 | (Zhao et al., 2011) |
| 1,3-dihydroxy-2-hydroxymethyl-anthraquinone -3-O-β-D-xylose-(1→6)-β-D-glycoside | NA | (Wang et al., 1992) |
| 1,3-dihydroxy-5,6-dimethoxy-2-(methoxymethyl)-anthraquinone | 1073237-09-8 | (Tuntiwachwuttikul et al., 2008) |
| 1,3-dihydroxy-5,6-dimethoxy-2-methyl anthraquinone | 872360-76-4 | (Feng et al., 2011) |
| 1,4-dihydroxy anthraquinone | 81-64-1 | (Liu et al., 2019) |
| 1,4-dihydroxy-2,3-dimethyl anthraquinone | 25060-18-8 | (Bidell et al., 1998) |
| 1,6-emodin dimethyl ether | 23610-20-0 | (Lin et al., 2015) |
| 1,7- dihydroxy-6-methoxy-2-methyl anthraquinone | 99520-76-0 | (Chen et al., 2016) |
| 1,8-​dihydroxy- 3-​[(acetyloxy)​methyl] ​ anthraquinone | 65615-58-9 | (Lin et al., 2015) |
| 1-[(β-D-glucopyranosyl-(1→3)-O-β-glucopyranosyl-(1→6)-O-β-D-glucopyranosyl) oxy]-8-hydroxy-3-methyl anthraquinone | 120181-07-9 | (Wong et al., 1989) |
| 1-[(β-D-glucopyranosyl-(1→6)-O-β-glucopyranosyl-(1→3)-O-β-D-glucopyranosyl-(1→6)-O-β-D-glucopyranosyl) oxy]-8-hydroxy-3-methyl anthraquinone | 120181-08-0 | (Wong et al., 1989) |
| 1-acetoxy-3-methoxy anthraquinone | 75299-65-9 | (Son et al., 2008) |
| 1-carbethoxy-3-methoxy anthraquinone | NA | (Son et al., 2007; Son et al., 2008) |
| 1-demethylaurantio-obtusin-2-O-β-D-glycopyranoside | 1159837-88-3 | (Tang et al., 2008) |
| 1-desmethylaurantio-obtusin | 90985-56-1 | (Chen et al., 2003) |
| 1-desmethylchryso-obtusin | 90985-58-3 | (Kitanaka and Takido, 1984) |
| 1-desmethylobtusin | 90985-57-2 | (Zhang et al., 2014) |
| 1-hydroxy anthraquinone | 129-43-1 | (Tanaka et al., 2000) |
| 1-hydroxy-2,3-dimethoxy-7-methyl anthraquinone | 872360-75-3 | (Feng et al., 2011) |
| 1-hydroxy-2-methyl anthraquinone | 6268-9-3 | (Verdan et al., 2015) |
| 1-hydroxy-3,7-diformyl anthraquinone | 255845-74-0 | (Chen et al., 2003) |
| 1-hydroxy-3-carbomethoxy anthraquinone | 133361-31-6 | (Koyama et al., 1992) |
| 1-hydroxy-3-ethoxy anthraquinone | 69008-00-0 | (Mishchenko et al., 2007) |
| 1-hydroxy-3-hydroxymethyl anthraquinone | 51995-90-5 | (Kang et al., 2017) |
| 1-hydroxy-4-methoxy anthraquinone | 7336-64-3 | (Chen et al., 2016) |
| 1-methoxy-2-methyl anthraquinone | 20460-44-0 | (Feng et al., 2011) |
| 1-O-methylemodin | 3775-08-4 | (Jia et al., 2009) |
| 2,3-dimethoxy-6-methyl anthraquinone | 105706-01-2 | (Chen et al., 2016) |
| 2,6-dihydroxy-3-methyl-4-methoxy anthraquinone | 1051940-52-3 | (Kang et al., 2008) |
| 2,6-dihydroxyanthraquinone | 84-60-6 | (Matsuda et al., 2001) |
| 2,7-dihydroxy-3-methyl anthraquinone | 1026501-91-6 | (Chen et al., 2016) |
| 2-acetyl-emodin | 32013-63-1 | (Lin et al., 2015) |
| 2-benzylx anthopurpurin | 34425-61-1 | (Zhang, 1992) |
| 2-formyl anthraquinone | 6363-86-6 | (Hou and Wan, 2008) |
| 2-formyl-3-hydroxy anthraquinone | 79212-87-6 | (Li and Jiang, 2018) |
| 2-hydroxy-1,3-dimethoxy anthraquinone | 19852-76-7 | (Chen et al., 2016) |
| 2-hydroxy-1-methoxy anthraquinone (alizarin-1-methylether) | 6170-06-5 | (Yang et al., 2019b) |
| 2-hydroxy-3-methoxy anthraquinone | 51439-86-2 | (Ma and Yang, 2019) |
| 2-hydroxy-3-methoxy-6-methyl anthraquinone | 1107660-68-3 | (Chen et al., 2016) |
| 2-hydroxy-3-methoxy-7-methyl anthraquinone | 885606-18-8 | (Chen et al., 2016) |
| 2-hydroxy-3-methyl anthraquinone | 17241-40-6 | (Nuñez Montoya et al., 2003) |
| 2-hydroxy-4-methoxy anthraquinone | 28504-24-7 | (Chen et al., 2016) |
| 2-hydroxy-6-methyl anthraquinone | 83312-50-9 | (Chen et al., 2016) |
| 2-hydroxyemodin-1-methylether | 346434-45-5 | (Yang et al., 2005) |
| 2-hydroxymethyl anthraquinone | 17241-59-7 | (Chen et al., 2016) |
| 2-hydroxymethyl-1-hydroxy anthraquinone | 24094-45-9 | (Chen et al., 2016) |
| 2-hydroxymethyl-3-hydroxy anthraquinone | 68243-30-1 | (Wu et al., 2009) |
| 2-methoxy anthraquinone | 3274-20-2 | (Feng et al., 2011) |
| 2-methoxy-1,3,6-trihydroxy anthraquinone | 871270-34-7 | (Chokchaisiri et al., 2019) |
| 2-methyl anthraquinone (tectoquinone) | 84-54-8 | (Chokchaisiri et al., 2019) |
| 2-methyl-3-methoxy anthraquinone | 17241-42-8 | (Chen et al., 2016) |
| 2-O-β-D-glucopyranosyl-nataloe-emodin | 113122-27-3 | (Conner et al., 1987) |
| 3-hydroxy-1,2-dimethoxy anthraquinone | 10383-62-7 | (Yang et al., 2019b) |
| 3-hydroxy-1,5,6-trimethoxy-2-methyl anthraquinone | 872360-77-5 | (Feng et al., 2011) |
| 3-hydroxymorindone | 80368-74-7 | (Zhang, 1992) |
| 4-hydroxy-1,2,3-trimethoxy-6-methyl anthraquinone | 1349075-21-3 | (Yang et al., 2019b) |
| 4-hydroxy-2-carboxy anthraquinone | 25186-77-0 | (Hou and Wan, 2008) |
| 5(8)-hydroxyalizarin-1-methylether | 34425-63-3 | (Feng et al., 2011) |
| 5-chlorosoranjidiol | 2095326-75-1 | (Zhang, 1992) |
| 5-hydroxydamnacanthol-ω-ethyl ether | 103956-44-1 | (Zhang, 1992) |
| 6'-acetyl- physcion -8-O-β-D-glycoside | NA | (Xian et al., 2017) |
| 6'-acetyl-emodin-8-O-β-D-glycoside | NA | (Xian et al., 2017) |
| 6-methoxyibericin | 168102-48-5 | (Zhang, 1992) |
| 8-hydroxysubspinosin | 80557-10-4 | (Zhang, 1992) |
| 8-O-methylchrysophanol (chrysophanol-8-methy ether) | 3300-25-2 | (Xian et al., 2017; Li and Jiang, 2018) |
| alaternin | 641-90-7 | (Paudel et al., 2020) |
| alaternin-1-O-β-D-glycopyranoside | 96820-53-0 | (Chen et al., 2003) |
| alaternin-2-O-β-D-glycopyranoside | 219607-92-8 | (Lee et al., 1998) |
| alizarin (1,2-dihydroxyanthraquinone) | 72-48-0 | (Badria et al., 2013) |
| alizarin 2-methyl ether | 6003-11-8 | (Son et al., 2008; Liu et al., 2016) |
| aloe-emodin | 481-72-1 | (Feng et al, 2014a) |
| aloe-emodin-8-O-(6'-O-acetyl)-glycoside | NA | (Ye et al., 2007) |
| aloe-emodin-8-O-(6'-O-galloyl)-glycoside | NA | (Sun et al., 2013; Sun et al., 2015) |
| aloe-emodin-8-O-β-D-glycoside | 33037-46-6 | (Sun et al., 2015) |
| aloesaponarin Ⅰ | 53254-89-0 | (Abdissa et al., 2017) |
| aloesaponarin Ⅱ | 53254-94-7 | (Abdissa et al., 2017) |
| anthrakunthone | 475673-79-1 | (Li and Jiang, 2018) |
| aurantio-obtusin | 67979-25-3 | (Hao et al., 2003) |
| aurantio-obtusin-6-O-β-D-glycopyranoside(glyco-aurantioobtusin) | 129025-96-3 | (Tang et al., 2008) |
| chrysarobin | 491-59-8 | (Chen et al., 2003) |
| chryso-obtusin | 70588-06-6 | (Xie et al., 2019) |
| chryso-obtusin 2-O-β-D-glycopyranoside | 96820-54-1 | (Chen et al., 2003) |
| chrysophanic acid-9-anthrone | 491-58-7 | (Acharya and Chatterjee, 1975) |
| chrysophanol | 481-74-3 | (Hao et al., 2003) |
| chrysophanol- 8-O-(6'-acetyl)-glycoside | NA | (Xian et al., 2017) |
| chrysophanol 8-O-(6'-O-galloyl)-glycoside | NA | (Ye et al., 2007) |
| chrysophanol-1-O-(6'-O-acetyl)-glycoside | NA | (Ye et al., 2007) |
| chrysophanol-1-O-β-D-gentiobioside | 54944-38-6 | (Chen et al., 2003) |
| chrysophanol-1-O-β-D-glycoside | 4839-60-5 | (Ye et al., 2007) |
| chrysophanol-8-O-(6'-O-malonyl)-glycoside | NA | (Yang et al., 1998) |
| chrysophanol-8-O-β-D-glycoside | 13241-28-6 | (Li and Zhang, 2016) |
| citreorosein (ω-hydroxyemodin) | 481-73-2 | (Lu et al., 2012) |
| cordifodiol | 604763-31-7 | (Abdullah et al., 2003) |
| cordifoliol | 1391565-28-8 | (Abdullah et al., 2003) |
| damnacanthal | 477-84-9 | (Chokchaisiri et al., 2019) |
| damnacanthol | 477-83-8 | (Dzoyem et al., 2016) |
| danthron (1,8-Dihydroxyanthraquinone) | 117-10-2 | (Zhou et al., 2005) |
| deoxyerythrolaccin | 18499-83-7 | (Lech et al., 2020) |
| digitolutein (2-hydroxy-3-methyl-1-methoxy anthraquinone) | 477-86-1 | (Zhang et al., 2008) |
| echinul polydric anthrone-8-O-D-glycopyranoside | NA | (Chen et al., 2003) |
| emodin | 518-82-1 | (Sun et al., 2015) |
| emodin anthrone | 491-60-1 | (Chen et al., 2003) |
| emodin-1-O-β-D-glycoside | 38840-23-2 | (Chen et al., 2014a) |
| emodin-1-O-β-gentiobioside | 849789-95-3 | (Tang et al., 2008) |
| emodin-3-diethyl ether | NA | (Chen et al., 1999) |
| emodin-6,8-dimethyl ether | 5018-84-8 | (Wang et al., 1996) |
| emodin-8-methyl ether (questin) | 3774-64-9 | (Zhang and Cui, 2016; Xian et al., 2017) |
| emodin-8-O-(6'-O-malonyl)-glycoside | NA | (Ye et al., 2007; Lo et al., 2015) |
| emodin-8-O-β-D-(6'-O-acetyl)-glycoside | 440087-84-3 | (Ye et al., 2007; Xian et al., 2017) |
| emodin-8-O-β-D-glycoside | 23313-21-5 | (Kim et al., 2008; Sun et al., 2015) |
| fallacinol | 569-05-1 | (Xian et al., 2017) |
| helminthosporin | 518-80-9 | (Augustin et al., 2020) |
| heterophylline | 482-91-7 | (Dimmer et al., 2017) |
| isoemodin | 476-62-0 | (Wu et al., 2010) |
| isoxanthorin | 65317-04-6 | (Noungoue et al., 2008) |
| knoxiadin | 98873-77-9 | (Zhao et al., 2011) |
| laccaic acid D | 18499-84-8 | (Hawas et al.,2006) |
| laccaic acid D-methylester | 53254-85-6 | (Van Wyk et al., 1995) |
| lucidin | 478-08-0 | (Yoo et al., 2010) |
| lucidinprimeveroside | 29706-59-0 | (Wang et al., 1992) |
| lucidin-ω-methylether (1,3-dihydroxy-2-methoxymethyl anthraquinone) | 79560-36-4 | (Hou and Wan, 2008) |
| majoronal | 34425-62-2 | (Zhang, 1992) |
| munjistin | 478-06-8 | (Gao et al., 2018) |
| nataloe-emodin | 478-46-6 | (Griffiths et al., 2016) |
| nordamnacanthal | 3736-59-2 | (Chokchaisiri et al., 2019) |
| obtusifolin | 477-85-0 | (Xie et al., 2019) |
| obtusifolin-2-O-β-D-(6'-O-acetyl) glycopyranoside | 1193512-20-7 | (Li et al., 2009a) |
| obtusifolin-2-O-β-D-glycoside | 120163-18-0 | (Wong et al., 1989) |
| obtusin | 70588-05-5 | (Mbatchou et al., 2017) |
| physcion | 521-61-9 | (Li and Zhang, 2016) |
| physcion-8-O-β-D-(6'-O-acetyl)-glycoside | 1956356-86-7 | (Sun et al., 2015; Xian et al., 2017) |
| physcion-8-O-β-D-glycoside | 23451-01-6 | (Kim et al., 2008; Sun et al., 2015) |
| physcion-d3 | 1215751-27-1 | (Lin et al., 2015) |
| prisconnatanone C | 2072112-78-6 | (Wang et al., 2015) |
| prisconnatanone D | 2072112-79-7 | (Wang et al., 2015) |
| prisconnatanone E | 2072112-80-0 | (Wang et al., 2015) |
| prisconnatanone F | 2072112-81-1 | (Wang et al., 2015) |
| prisconnatanone G | 2072112-82-2 | (Wang et al., 2015) |
| prisconnatanone H | 2072112-83-3 | (Wang et al., 2015) |
| prisconnatanone I | 2072112-84-4 | (Wang et al., 2015) |
| pseudopurpurin | 476-41-5 | (Wu et al., 2012) |
| purpurin | 81-54-9 | (Gao et al., 2018) |
| pustuline | 885606-17-7 | (Dimmer et al., 2017) |
| quinalizarin | 81-61-8 | (Zang et al., 2019) |
| rhein | 478-43-3 | (Xiao et al., 2020) |
| rhein-1-O-(O-acetyl)-glycoside | NA | (Ye et al., 2007) |
| rhein-1-O-β-D-glycoside | NA | (Ye et al., 2007) |
| rhein-8-O-β-D-glycoside | 34298-86-7 | (Ye et al., 2007) |
| ruberitrinic acid | 50764-64-2 | (Mishchenko et al., 2007) |
| ruberythric acid | 152-84-1 | (Ford et al., 2015) |
| rubiacordone A (6-acetoxy-1-hydroxy-2-methylanthraquinone-3-o-alpha-l-rhamnopyranoside) | 1123303-25-2 | (Li et al., 2009b) |
| rubiadin | 117-02-2 | (Peng et al., 2017) |
| rubiadin-1-methyl ether | 7460-43-7 | (He et al., 2018) |
| rubiasin A | NA | (Leng et al., 2000) |
| rubiasin B | NA | (Leng et al., 2000) |
| rubiasin C | NA | (Leng et al., 2000) |
| sorandidiol | 518-73-0 | (Son et al., 2008) |
| soranjidiol | 79820-25-0 | (Son et al., 2008; Dimmer et al., 2017) |
| subspinosin | 80565-16-8 | (Zhang, 1992) |
| xanthopurpurin (purpuroxanthine) | 518-83-2 | (Zhao et al., 2011; Han et al., 2020) |
| ω-hydroxyemodin-8-methyl ether (questinol) | 35688-09-6 | (Liu et al., 2009) |
| **Dinuclear anthraquinones** |  |  |
| 5,5’-bisoranjidiol | 885024-41-9 | (Dimmer et al., 2017) |
| 7-chlorobisoranjidiol | 2095326-74-0 | (Dimmer et al., 2017) |
| bianthrone A | 116-71-2 | (Mbwambo et al., 2004) |
| cassiamin A | 98332-81-1 | (Li and Jiang, 2018) |
| cassiamin B | 27567-10-8 | (Li and Jiang, 2018) |
| chrysophanol-10,10'-bianthrone | 17062-54-3 | (Chen et al., 2003) |
| emodin bianthrones A (trans-emodin dianthrones) | 61281-20-7 | (Xian et al., 2017) |
| emodin bianthrones B (cis -emodin dianthrones) | 61281-19-4 | (Xian et al., 2017) |
| hypericin | 548-04-9 | (Laub et al., 2020) |
| lycionine | NA | (Dimmer et al., 2017) |
| madagascarin | 36506-89-5 | (Li and Jiang, 2018) |
| morindaquinone | NA | (Chokchaisiri et al., 2019) |
| palmidin A | 17062-55-4 | (Luo et al., 2017) |
| polygonumnolide E | NA | (Yang et al., 2017) |
| polygonumnolides A1 | 2133040-28-3 | (Yang et al., 2018b) |
| polygonumnolides A2 | 2133040-27-2 | (Yang et al., 2018b) |
| polygonumnolides A3 | 2133040-29-4 | (Yang et al., 2018b) |
| polygonumnolides A4 | 2133040-30-7 | (Yang et al., 2018b) |
| polygonumnolides B1 | 2133040-33-0 | (Yang et al., 2018b) |
| polygonumnolides B2 | NA | (Yang et al., 2018b) |
| polygonumnolides B3 | 2133040-31-8 | (Yang et al., 2018b) |
| polygonumnolides C1 | NA | (Yang et al., 2016b) |
| polygonumnolides C2 | NA | (Yang et al., 2016b) |
| polygonumnolides C3 | NA | (Yang et al., 2016b) |
| polygonumnolides C4 | NA | (Yang et al., 2016b) |
| rheidin A | 959582-45-7 | (Gu et al., 2011) |
| rheidin C | 959573-85-4 | (Gu et al., 2011) |
| sennidine A | 641-12-3 | (Waltenberger et al., 2008) |
| sennidine B | 517-44-2 | (Waltenberger et al., 2008) |
| sennidine C | 5355-93-1 | (Waltenberger et al., 2008) |
| sennoside A | 81-27-6 | (Hwang and Jeong, 2015; Shah et al., 2000) |
| sennoside B | 128-57-4 | (Hwang and Jeong, 2015; Shah et al., 2000) |
| sennoside C | 37271-16-2 | (Gu et al., 2011; Shah et al., 2000) |
| sennoside D | 37271-17-3 | (Ha et al., 2012; Shah et al., 2000) |
| sennoside E/F | 52842-23-6 | (Hiraoka et al., 1989) |

Note: NA: not available.
